# Diet at Age 10 and 13 Years in Children Identified as Picky Eaters at Age 3 Years and in Children Who Are Persistent Picky Eaters in A Longitudinal Birth Cohort Study

**DOI:** 10.3390/nu11040807

**Published:** 2019-04-10

**Authors:** Caroline M Taylor, Nicholas P Hays, Pauline M Emmett

**Affiliations:** 1 Centre for Academic Child Health, Bristol Medical School, University of Bristol, Bristol BS8 1NU, UK; P.m.emmett@bristol.ac.uk; 2 Nestlé Product Technology Center—Nutrition, La Tour-de-Peilz, 1800 Vevey, Switzerland; NicholasPaul.Hays@nestle.com

**Keywords:** ALSPAC, child, nutrition, picky eater, food neophobia, fussy eater, food avoidance

## Abstract

Picky eating has been associated with lower intakes of some nutrients and foods during preschool ages but there is little known about the longer-term diet. The aim of this study was to characterise the diets of children aged 10 and 13 years who had been identified as: (1) picky eaters at age 3 years (cross-sectional); and (2) picky eaters at 2–5.5 years old (longitudinal). Picky eating behaviour (PE) was identified in the Avon Longitudinal Study of Parents and Children (ALSPAC) from parental/caregiver questionnaires. Dietary intake was assessed at age 3.5 years and repeated at 10 and 13 years. For cross-sectional PE compared with non-PE there were differences at age 10 years that were similar to those at 3.5 years: lower intakes of protein (−5%) and fibre (−7%) and of meat (−15%), fruit (−10%) and vegetables (−33%). At 13 years, differences in vegetable (−23%), fruit (−14%) and meat (−8%) intakes were evident. For longitudinal (persistent) PE, differences were more pronounced at each age. More effective strategies to help parents to widen the food choices of their children at early ages need to be developed, focusing particularly on vegetable and fruit intakes.

## 1. Introduction

While there is no universally accepted definition for the term ‘picky eating,’ this behaviour is often characterised as an unwillingness to eat familiar foods or to try new foods, as well as strong food preferences [1,2]. The prevalence has been estimated at 6–60% depending on the method of assessment and categorisation, age of child, and the country in which the study is conducted [1], with peak occurrence at about 3 years old [1]. Picky eating has been associated with a reduction in dietary variety and consequently an unhealthy or possibly inadequate diet [3,4,5], with picky eaters eating a smaller range of food items than non-picky eaters and having lower diversity and variety scores [3,4,5,6]. In turn, this can result in being underweight and having poor growth [7,8,9,10,11,12,13,14,15,16] or being overweight [17], having gastrointestinal disorders [18,19] and possibly developing eating disorders [20].

Picky eating in young children may result in alterations in the intakes of energy [21] and some micronutrients [4,15,16,22,23]. The latter is driven mainly by reductions in vegetable intake and to a lesser extent, fruit consumption [6,15,16,22,23,24,25,26,27,28,29]. Lower consumption of wholegrain products, fish/seafood and meat and unsweetened cereals and higher consumption of savoury snacks and confectionary, sweetened cereals and French fries in picky eaters compared with non-picky eaters have also been noted [26,27]. However, most of these studies have focussed on dietary intakes in preschool children, so the effects extending later into childhood are unknown [1], with the exception of low fruit and vegetable intakes in girls at early adolescence who were persistent picky eaters [30]. To our knowledge there are no longitudinal studies of dietary intakes in older children or adolescents who were identified as picky eaters in preschool years. Information on the persistence of differences in dietary intake associated with picky eating in early childhood could assist in evaluation of the effect on long-term nutrition and health outcomes and facilitate the development of advice to parents and health professionals on the management of picky eating. 

Within a longitudinal cohort of children from the UK Avon Longitudinal Study of Parents and Children (ALSPAC), we have previously characterised a group of children who were identified as picky eaters at 3 years old [1], as well as carrying out a longitudinal assessment of picky eating over four ages between 2 and 5.5 years old [23]. The results of analyses of dietary intakes at age 3.5 years comparing *very*, *somewhat* and *non-picky* children have been reported [23]: picky eaters had lower mean protein, carotene, iron and zinc intakes than non-picky eaters, without any difference in energy intake. Nutrient differences were explained by lower intakes of meat, fish, vegetables and fruits in picky eaters than in non-picky eaters. When we investigated longitudinal picky eating, three main categories of picky eating were defined (*non-persistent*, *persistent* and *late-onset* [23]). When dietary intakes at age 7 years were compared with those of *never* picky children, similar nutrients and food groups to those at 3.5 years were affected. Differences were generally largest in the *persistent* and *late-onset* groups. Furthermore, there was evidence that children in all the picky-eater groups ate more free sugars on average than *never* picky children at age 7 years.

The aim of the present study was to extend these analyses to characterise the diets of picky children at older time points (age 10 and 13 years) and compare them with the diets of: (1) *non-picky* children (cross-sectional classification at age 3 years); (2) *never* picky children (longitudinal classification age 2–5.5 years). Differences in food group and nutrient intakes between the picky eating groups are compared across the ages. 

## 2. Methods

ALSPAC is a longitudinal population-based study investigating environmental and genetic influences on the health, behaviour and development of children. It has covered diet extensively and collected questionnaire data from parents about feeding their child.

All pregnant women in the former Avon Health Authority in south-west England with an expected delivery date between 1 April 1991 and 31 December 1992 were eligible for the study; 14,541 pregnant women were enrolled, resulting in a cohort of 14,062 live births with 13,988 alive at 1 year of age [31,32]. Further details of ALSPAC are available at www.bris.ac.uk/alspac and the study website contains details of all the data that are available through a fully searchable data dictionary (http://www.bris.ac.uk/alspac/researchers/data-access/data-dictionary). Ethics approval for the study was obtained from the ALSPAC Ethics and Law Committee and the Local Research Ethics Committees.

### 2.1. Defining Picky Eating Cross-sectionally (at 3 Years) and Longitudinally (2–5.5 Years) in the ALSPAC Cohort

The primary caregiver (usually the mother) received postal self-completion questionnaires when their child was 3 years old. The questionnaires are available from the study website (http://www.bristol.ac.uk/alspac/researchers/questionnaires/). Singletons only (of any birth order) were selected for all phenotype definitions. A single question similar to those used in several recent studies [33,34,35,36] was asked.

Q. Does your child have definite likes and dislikes as far as food is concerned? 

A. No/Yes, quite choosy/Yes, very choosy

### 2.2. Cross-sectional Classification

The responses were scored 0, 1 or 2, respectively. For the categorisation of picky eating at age 3 years, *very picky* eaters were defined as those who scored 2, *non-picky* as those who scored 0 and those scoring 1 as *somewhat picky*. 

### 2.3. Longitudinal Classification

**Figure 1:**
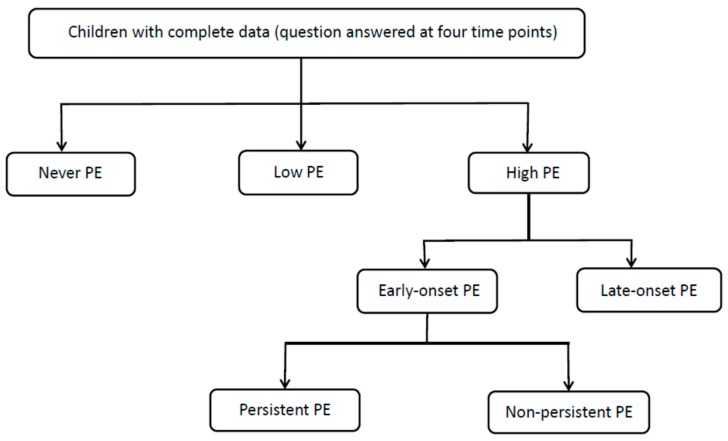
Chart to show longitudinal classification of picky eating in children aged 2–5.5 years enrolled in the Avon Longitudinal Study of Parents and Children (ALSPAC).

The same question was asked about the child at three additional timepoints (2, 4.5 and 5.5 years) and the answers were coded in the same way. For the longitudinal classification, *never* picky eating was defined as a score of 0 at all time points; *low* picky eating as a score of 1 at all time points or 0 or 1 at three points and 2 at any one time point; *high* picky eating as a score of 2 at two or more time points. *High* picky eating was further subdivided into *early-onset* (score of 2 first occurring at either age 2 or 3 years) or *late-onset* (score of 2 first occurring at age 4.5 or 5.5 years). *Early-onset* picky eating was further subdivided in to *persistent* (score 2 at least three times between 2 and 5.5 years and *non-persistent* (did not score 2 after the age of 3 years) [23] (Figure 1).

### 2.4. Dietary Assessment

As reported previously [23], a convenience subsample of 10% of the ALSPAC cohort was invited to a research clinic when the children were 3.5 years of age. Prior to the clinic parents were mailed a structured diary to record all the foods and drinks that the child consumed over three individual days (one weekend day and two weekdays) in household measures. The food records (FR) were checked with the parents in the clinic and then used to calculate daily mean nutrient intakes for each child, as described by Emmett, et al. [37]. In brief, the records were coded and analysed using an in-house nutrient analysis program based on the fifth edition of McCance and Widdowson’s food composition tables and supplements to the tables (cited in Emmett, Rogers, Symes and Team [37]). Free sugars were defined as all monosaccharides and disaccharides added to foods by the manufacturer, cook or consumer, plus sugars naturally present in honey, syrups, fruit juices and fruit juice concentrates. A similar method of dietary data collection and analysis was used when the children were 7, 10 and 13 years of age but the whole ALSPAC cohort of children was invited to attend. At 10 and 13 years the children themselves recorded their intake with help from their parents. To improve accuracy the child and parents were interviewed about the FR by a trained fieldworker during a research clinic session. 

Misreporting of dietary data, which is a common problem in dietary assessment and can lead to under- or over-estimates of energy and nutrient intakes, was assessed and classified at age 10 and 13 years using an individualised method based on predicted energy requirement that takes into account the age, sex and body weight of the child and allows for growth and a standard level of physical activity [38]. The ratio of reported energy intake (EI) to estimated energy requirement (EER) was calculated (EI:EER). Individual EERs were estimated using equations from an *Expert Consultation Report on Human Energy Requirements* [39]. A 95% confidence interval for the accuracy of EI:EER was calculated by taking into account the amount of variation inherent in the methods used to estimate EI and EER [40]. For example, the confidence range for EI:EER calculated for the data at age 7 years was 0.79–1.21, so reports of EI between 79% and 121% of EER were considered to be within the normal range of measurement error and were defined as plausible reports. Those below this cut-off were under-reports and those above were over-reports [41].

### 2.5. Statistical Analysis

Statistical analyses were carried out with SPSS v23 (IBM Corp.). Statistical comparisons were made between the different categories of picky eaters (analysis of variance with a multiple comparison correction (Bonferroni)). Mean nutrient intakes and weights consumed of each food group (see Supplementary Text S1) were compared between picky eating groups. In general, only those nutrients and food groups with statistically significant differences in intakes between picky eating groups are presented in the tables. For micronutrients for which differences between the groups persisted to age 13 years, the percentage of children with intakes below the UK lower reference nutrient intake (LRNI) [42] were calculated. The analyses were then repeated for children with plausible energy intakes only.

## 3. Results

### 3.1. Cross-Sectional

In the cross-sectional analysis with picky eating categorised at 3 years of age, lower intakes of protein, dietary fibre, carotene, iron, zinc and selenium in *very picky* eaters than in the *non-picky* eaters have previously been identified at age 3.5 years [23]. At age 10 years, intakes of these nutrients were again lower in the *very picky* group than in the *non-picky* group, with the exception of iron (Table 1). By age 13 years, only lower mean zinc intake was evident. However, at both 10 and 13 years of age *somewhat* and *very picky* eaters had higher mean intakes of free sugars than *non-picky* children, although this higher intake had not been observed at 3.5 years. When zinc intakes for each group at age 10 years were assessed against the UK LRNI of 4.0 mg zinc/day for this age [42], 9.0% of *very picky*, 6.1% of *somewhat picky* and 5.9% of *non-picky* children had intakes at or below this amount (*non-picky* vs. *somewhat picky p* = 0.865, *non-picky* vs. *very picky p* = 0.003).

Except for total milk intake, differences in food group intakes observed at age 3.5 years were retained at age 10 years, with *very picky* eaters eating less total and carcass meat, fish, vegetables, fruit and savoury sauces (gravy, salad dressing, barbeque sauce, etc.) than *non-picky* children (Table 2). By 13 years, there were fewer differences: lower total meat, vegetables, fruit and savoury sauces only. Conversely at age 10 years, intakes of some sweet foods (chocolate confectionary, sweet biscuits and cookies) were greater in *very picky* eaters than *non-picky* eaters (by 24% and 11%, respectively). Greater intake of chocolate confectionary was also evident at 13 years. 

### 3.2. Longitudinal

On investigating the diets of the longitudinally-defined picky eating groups at 10 years, differences in nutrient intakes compared with the *never* group were most evident in the *persistent* group (for example, protein 10% lower, free sugar 9% higher, dietary fibre 13% lower, retinol equivalents 17% lower, zinc 14% lower, iron 8% lower) (Table 3). There were some small differences for *low* (for example, protein 4% lower, retinol equivalents 6% lower, dietary fibre 3% lower), even fewer for *late* (only protein and fibre, 8% lower and 10% respectively) and none for *non-persistent* compared with the *never* group. The proportion of children in each group with intakes of zinc below the LRNI were 5.5%, 6.2%, 6.5%, 10.2% and 10.2% for *never*, *low*, *non-persistent*, *persistent* and *late* groups, respectively (*never* vs. *low p* = 0.230, *never* vs. *non-persistent p* = 0.497, *never* vs. *persistent p* < 0.001, *never* vs. *late p* = 0.055). At age 13 years these differences had disappeared except for two nutrients in the *persistent* group (protein 5% lower and free sugar 11% higher) and one for the *low* group (free sugar 5% higher).

For the food group analyses the most striking finding was that at both ages all four picky eating groups had lower mean intakes of vegetables than the *never* picky children (age 10 years 15–49% lower; age 13 years 10–43% lower) (Table 4). Intakes of carcass meat were also consistently lower in the picky eating groups at age 10 years than in the *never* group (10–37% lower) but less so at 13 years when the only difference was for the *late* group (34% lower). The group with the most dietary differences overall compared with the *never* picky children at both ages was *persistent*. 

### 3.3. Effect of Misreporting

Misreporting at ages 10 and 13 years varied with picky eating status: picky children were less likely to under-report their intake than non-picky children (Supplementary Table S1). In general, when the diets of children with plausible intakes only were assessed, the overall finding of dietary differences being retained at age 10 years and largely resolving by age 13 years was similar. For the cross-sectional analysis, in children with plausible energy intakes only, at 10 years there was evidence of slightly lower energy intakes in picky compared with non-picky children but by 13 years these differences were not evident (Supplementary Table S2). Other nutrient and food group differences were very similar to those in the whole cohort (Supplementary Tables S2 and S3). For the longitudinal analyses, in children with plausible intakes only, there was evidence of lower energy intake in some picky groups compared with the *never* group (*low* –−0.28 MJ/day; *persistent* −0.34 MJ/day; *late* −0.44 MJ/day; Supplementary Table S4). These differences were related to lower intakes of both protein and fat and were not seen in the whole sample. The *persistent* group showed the lowest micronutrient intakes. These differences had resolved by 13 years although lower intakes of fruit, vegetables and savoury sauces remained evident in the *persistent* group (Supplementary Tables S4 and S5).

## 4. Discussion

In the group of children in the ALSPAC study who were identified as *very picky* eaters or *non-picky* eaters at age 3 years, there were differences between the groups in the dietary intakes of some nutrients (protein, free sugar, carotene, zinc, selenium) and food groups (total meat, carcass meat, fish, vegetables, fruit) at age 3.5 years that were still evident at age 10 years. By age 13 years these differences were tending to disappear but there were still differences in the intakes of total meat, vegetables and fruit. Differences in intakes of zinc were evident at each of the three ages (3.5, 7 and 10 years). There were no differences in energy intake between *somewhat* or *very picky* eaters and *non-picky* eaters at any of the three ages, except when only plausible reporters were assessed. In plausible reporters there were slightly lower mean energy intakes in *somewhat* or *very picky* eaters compared with *non-picky* at age 10 years but not at 13 years. Overall nutrient difference between groups were small and not likely to have any physiological relevance. However, the low fruit and vegetable intake apparent in most of the children is a marker of poor diet quality.

When picky eating status was assessed longitudinally, children who were *persistent* picky eaters between age 2 years and 5.5 years showed the most consistent nutrient differences at age 10 years from those who were *never* picky. Again, most of the differences had resolved by age 13 years, although lower protein and higher free sugars intakes remained. Food group intake differences between the *never* picky children and all the picky eating groups were evident at both ages particularly lower vegetable intakes. However, the *persistent* picky eaters showed the greatest number of differences in food group intakes at 10 years (lower carcass and total meat, potatoes, vegetables and savoury sauces and higher chocolate and sweet biscuit intakes), most of which were still evident at age 13 years. All children had adequate protein intakes but the diets of persistent picky eaters were of lower quality due to lower vegetable and higher free sugar intakes.

Studies of picky eaters are usually of cross-sectional design and so have not been able to explore the long-term effects on dietary intakes. To our knowledge, this is the first study in which diet in picky eaters has been reported at more than one time point. It also provides dietary data at the oldest age (13 years) reported to date (the oldest age group in previous reports to our knowledge is age 9 years in girls only [22]). 

The persistence of dietary differences into early adolescence could arise for a number of reasons. There is evidence that some picky eaters are likely to remain as picky eaters into young adulthood. Van Tine, et al. [43], for example, followed up 61 young adults who had been identified as ‘selective eaters’ (used synonymously with picky eating) between age 2 and 11 years old: of the 10 children who were identified as being selective at age 3 years, the same age as in our study, six were still selective eaters at age 23 years. However, of the 51 who were not selective eaters at age 3 years, 11 had become selective eaters by age 23, suggesting an emerging ‘late onset’ group. In addition, those who were selective eaters for more than 6 years before the age of 11 remained selective at age 23, suggesting a further pattern of ‘persistent’ selectivity. There was no evidence of increased psychopathology associated with eating disorders, excessive thinness or obesity in the selective eaters compared with the non-selective eaters. We do not have any information about the picky eating status of our participants beyond age 5.5 years and particularly on whether picky eating emerged in some of those who were never picky before this age: it is possible that there is underlying ‘misclassification’ at age 13 years because of this but our primary intention was to look at associations with classification at a single early timepoint. It is also possible that some of participants at age 13 years who were classified as picky eaters at age 3 years had emerging eating disorders: evidence for an effect of being a picky eater in early childhood on the development of later eating disorders is conflicting, with some authors reporting an association [20] but others not [44]. Strategies for parents to help their children overcome picky eating before it becomes persistent include repeated exposure of food, having realistic expectations of portion sizes, having a positive approach, not providing snacks between meals or letting the child drink excessively and providing social food experiences and consistency [45].

The present study and other similar but cross-sectional studies show that picky eating can result in unhealthy or sometimes inadequate diets [4,15,16,22,26], with potential adverse health consequences [9,18,19,46]. It is well established that picky eaters have low intakes of fruit and vegetables [6,15,16,22,23,24,25,26,27,28,29]; the reason for this is not clear but may be related to a dislike of certain textures or tastes, possibly related to bitterness [47]. Although there appears to be a genetic predisposition to pickiness [48], repeated offerings of fruits and vegetables, as well as parental modelling, are very important in overcoming picky behaviour [49,50]. There are several reports of lower meat intakes than in non-picky eaters [23,27] and this may be related to late introduction of lumps (chewy foods) during weaning [50,51]. 

There are several strengths in this study as described previously [23]: (1) the definition of picky eating using an unambiguous question about child choosiness that did not invite the parents to define picky eating for themselves; (2) the inclusion of non-picky comparison groups in both analyses; (3) the use of FR with household measures, a well-respected method to assess diet rather than the less precise food frequency questionnaire (FFQ) method [52]; and (4) the opportunity to assess picky eating over time; (5) the inclusion of analyses of all cases data, which enables comparisons to be made with other studies that report data in this way, is enhanced by inclusion of analyses of data from plausible reporters only giving an indication of the reliability of the results. Limitations include: (1) picky eating categorisation was based on a single question and did not cover the full range of ‘picky eating’ traits as defined in some other studies; (2) identification of avoidant/restrictive food intake disorder (ARFID; previously known as selective eating disorder) according to the DSM-5 definition was beyond the scope of the present study and was not possible within the constraints of the data available; (3) the relatively small numbers of children in some of the picky eating status groupings (this could affect the assumption of homogeneity of variance for ANOVA); (4) attrition and incomplete data collection is inevitable in longitudinal studies; here 60% of the 10% sub-sample provided FR at 3.5 years and 55% of the original cohort provided them at 10 years and 45% at 13 years [53].

## 5. Conclusions

The differences in food and food group intakes and in nutrient intakes evident at 3.5 years between children who were picky eaters or not at age 3 years showed evidence of persisting into adolescence particularly for vegetable, fruit and meat intakes. The nutritional relevance or health impact of these differences requires further research. The development of more effective strategies focused on helping parents to widen the food choices of their children at an early age need to be developed. These strategies should focus particularly on vegetable and fruit intakes.

## Figures and Tables

**Table 1:** Nutrient intakes from 3-day food records at age 10 and 13 years in children enrolled in ALSPAC who were classified into picky eating categories at age 3 years (cross-sectional).

Nutrient	Age 10 Years			Age 13 Years		
	Non-picky	Somewhat Picky	Very Picky	Non-picky	Somewhat Picky	Very Picky
*n*	2341	2169	804	1981	1820	693
Macronutrients						
Protein (g/day)	62.9 (62.2, 63.5)	61.7 (61.0, 62.3) *	59.5 (58.4, 60.5) ***	69.3 (68.0, 69.9)	68.9 (68.0, 69.9)	67.9 (66.3, 69.5)
Free sugar (% energy)	17.3 (17.1, 17.5)	18.0 (17.7, 18.2) ***	18.2 (17.8, 18.6) ***	16.4 (16.1, 16.7)	17.0 (16.7, 17.3) *	17.2 (17.0, 18.0) ***
Dietary fibre (g/day)	11.7 (11.5, 11.8)	11.5 (11.3, 11.7)	10.9 (10.6, 11.1) ***	12.9 (12.7, 13.1)	13.1 (12.8, 13.2)	12.5 (12.1, 12.8)
Micronutrients						
Vitamin A						
Carotene (µg/day)	2135 (2068, 2202)	2086 (2013, 2158) ***	1863 (1740, 1985) ***	2376 (2285, 3467)	2360 (2264, 2457)	2356 (2193, 2420)
Vitamin B_12_ (µg/day)	3.47 (3.40, 3.64)	3.41 (3.34, 3.48)	3.35 (3.21, 3.48)	4.43 (4.32, 4.54)	4.21 (4.10, 4.53) *	4.23 (4.05, 4.40)
Vitamin D (µg/day)	2.65 (2.60, 2.71)	2.67 (2.60, 2.73)	2.47 (2.38, 2.56) **	2.69 (2.62, 2.75)	2.66 (2.59, 2.73)	2.61 (2.49, 2.73)
Vitamin E (mg/day)	9.28 (9.14, 9.43)	9.15 (9.00, 9.31)	8.85 (8.58, 9.13) *	9.29 (9.10, 9.47)	9.15 (8.96, 9.33)	8.96 (8.64, 9.28)
Zinc (mg/day)	6.9 (6.7, 6.9)	6.7 (6.7, 6.8)	6.4 (6.3, 6.6) ***	7.6 (7.5, 7.8)	7.5 (7.4, 7.6)	7.3 (7.1, 7.5) *
Iron (mg/day)	9.0 (8.9, 9.1)	9.0 (8.9, 9.1)	8.8 (8.8, 9.0)	10.0 (9.9, 10.2)	10.2 (9.9, 10.2)	10.0 (9.9, 10.1)
Selenium (µg/day)	58.3 (57.5, 59.1)	57.8 (57.0, 58.7)	55.7 (54.3, 57.1) **	62.6 (61.3, 63.8)	62.9 (61.7, 64.0)	62.1 (60.0, 64.2)

Data at age 3.5 years are shown in Taylor, Northstone, Wernimont and Emmett [23] and Taylor, Northstone, Wernimont and Emmett [19]. Values are mean (95% confidence interval). Value at same age significantly different from that of children who were non-picky eaters: * *p* ≤ 0.05, ** *p* ≤ 0.01, *** *p* ≤ 0.001 (corrected for multiplicity). Additional nutrients for which there were no significant differences at both 10 and 13 years (data not shown): energy (for males and females separately and for the whole group), carbohydrate, fat, retinol, retinol equivalents, thiamin, riboflavin, niacin, vitamin B_6_, vitamin B_12_, folate, vitamin C, calcium, iodine.

**Table 2:** Food and food group intakes (g/day) from 3-day food records at age 10 and 13 years in children enrolled in ALSPAC who were classified into picky eating categories at age 3 years (cross-sectional).

Food and Food Group Intake (g/day)	Age 10 Years			Age 13 Years		
	Non-Picky	Somewhat Picky	Very Picky	Non-Picky	Somewhat Picky	Very Picky
*n*	2341	2169	804	1981	1820	693
Total meat	107 (104, 110)	99 (97, 102) ***	91 (87, 95) ***	125 (122, 129)	119 (115, 123)	115 (109, 122) *
Meat, carcass ^1^	60 (58, 62)	56 (53, 58) *	44 (41, 48) ***	76 (73, 79)	72 (69, 76)	69 (64, 75)
Processed meat ^2^	47 (45, 49)	44 (42, 45) *	47 (44, 50)	49 (47, 52)	47 (44, 49)	46 (43, 50)
Total fish	14 (13, 15)	14 (13, 15)	11 (10, 13) *	16 (15, 17)	17 (15, 18)	14 (12, 16)
Total vegetables	75 (73, 78)	67 (65, 70) ***	50 (47, 54) ***	91 (88, 94)	85 (82, 89)	70 (65, 75) ***
Total fruit	72 (69, 75)	70 (67, 73)	63 (58, 68) *	80 (76, 84)	80 (76, 85)	69 (63, 76) *
Total milk ^3^	214 (207, 221)	211 (203, 219)	226 (212, 240)	224 (214, 234)	208 (199, 218)	228 (211, 244)
Total sweet foods	126 (123, 129)	129 (126, 132)	124 (120, 129)	106 (103, 110)	107 (104, 111)	115 (109, 121) *
Chocolate confectionery	17 (16, 18)	19 (18, 20) *	21 (19, 22) ***	16 (15, 17)	17 (16, 18)	20 (18, 22) ***
Buns, cakes and pastries	27 (26, 29)	29 (27, 30)	26 (24, 28)	26 (24, 28)	28 (26, 29)	28 (25, 31)
Sweet biscuits and cookies	19 (18, 20)	19 (18, 20)	21(20, 23) *	18 (17,19)	19 (18, 20)	19 (18, 20)
Carbonated drinks with sugar	105 (99, 112)	99 (93, 106)	108 (97, 119)	128 (119, 137)	127 (117, 137)	135 (117, 152)
Fruit juice	114 (108, 120)	126 (119, 133) *	122 (111, 133)	153 (144, 162)	178 (168, 188) **	173 (157, 189)
Savoury sauces ^4^	16 (15, 16)	13 (13, 14) ***	12 (11, 13) ***	18 (17, 19)	16 (15, 17) *	15 (13, 16) **

Data at age 3.5 years are shown in Taylor, Northstone, Wernimont and Emmett [23] and Taylor, Northstone, Wernimont and Emmett [19]. Values are mean (95% confidence interval). Value at same age significantly different from that of children who were not picky eaters: * *p* ≤ 0.05, ** *p* ≤ 0.01, *** *p* ≤ 0.001 (corrected for multiplicity). ^1^ Foods included carcass meat from lamb, pork, beef, poultry, liver and kidney. ^2^ Foods included sausages, ham, bacon, burgers, meat pies, breaded poultry, salami, etc. ^3^ Milks included whole, semi-skimmed, skimmed cows’ milk, other animal milks, soya milk, formula milk and cream. ^4^ Savoury sauces included gravy, salad dressing, barbecue sauce, etc.

**Table 3:** Nutrient intakes from 3-day food records at age 10 and 13 years in children enrolled in ALSPAC who were classified into longitudinal picky eating (PE) categories between 2 and 5.5 years of age.

Nutrient	Never PE	Low PE	High PE		
			Early PE		Late PE
			Non-Persistent PE	Persistent PE	
Age 10 years					
*n*	1380	3159	275	402	98
Macronutrients					
Energy (kJ/day)	7927 (7834, 8015) ^a^	7771 (7717, 7825) ^b^	7901 (7715, 8087) ^a,b^	7688 (7536, 7840) ^a,b^	7600 (7295, 7905) ^a,b^
Male	8353 (8222, 8484) ^a^	8175 (8099, 8251) ^a,b^	8277 (8003, 8551) ^a,b^	7935 (7731, 8138) ^b^	8233 (7747, 8719) ^a,b^
Protein (g/day)	63.9 (63.1, 64.8) ^a^	61.6 (61.0, 62.1) ^b^	61.5 (59.8, 63.3) ^a,b^	57.8 (56.3, 59.3) ^c^	59.1 (56.0, 63.3) ^b,c^
Fat (g/day)	77.1 (76.0, 78.2) ^a^	74.9 (74.3, 75.6) ^b^	76.2 (73.8, 78.5) ^a,b^	74.1 (72.3, 76.1) ^a,b^	72.6 (68.7, 76.4) ^a,b^
Free sugar (% energy)	17.1 (16.8, 17.4) ^a^	17.8 (17.6, 18.0) ^b^	17.8 (17.1, 18.5) ^a,b^	18.6 (18.0, 19.2) ^b^	18.7 (17.6, 19.9) ^a,b^
Dietary fibre (g NSP/day)	11.9 (11.7, 12.1) ^a^	11.5 (11.3, 11.6) ^b^	11.3 (10.9, 11.7) ^a,b^	10.4 (10.0, 10.7) ^c^	10.7 (10.0, 11.3) ^b,c^
Micronutrients					
Vitamin A					
Carotene (µg/day)	2244 (2053, 2336) ^a^	2054 (1997, 2112) ^b^	2052 (1856, 2247) ^a,b,c^	1694 (1515, 1874) ^c^	1902 (1452, 2355) ^a,b,c^
Retinol equivalents (µg RE/day)	738 (713, 763) ^a^	692 (675, 709) ^b^	767 (684, 850) ^a,b^	609 (574, 644) ^c^	767 (684, 851) ^a,b,c^
Niacin (mg NEq/day)	30.0 (29.6, 30.4) ^a^	29.2 (29.0, 29.5) ^b^	29.5 (28.6, 30.3) ^a,b,c^	28.2 (26.6, 29.8) ^c^	29.3 (29.1, 29.5) ^a,b,c^
Vitamin B_6_ (mg/day)	1.9 (1.9, 1.9) ^a^	1.9 (1.8, 1.9) ^a^	1.9 (1.8, 2.0) ^a^	1.8 (1.7, 1.8) ^b^	1.8 (1.7, 1.9) ^a,b^
Vitamin B_12_ (µg/day)	3.5 (3.4, 3.6) ^a^	3.4 (3.4, 3.5) ^a,b^	3.5 (3.2, 3.8) ^a,b^	3.2 (3.0, 3.3) ^b^	3.5 (3.0, 4.0) ^a,b^
Folate (µg/day)	214 (210, 218) ^a^	208 (205, 210) ^a,b^	200 (192, 207) ^a,b^	215 (206, 223) ^b^	195 (182, 208) ^a,b^
Vitamin D (µg/day)	2.7 (2.6, 2.8) ^a^	2.6 (2.6, 2.7) ^a^	2.6 (2.4, 2.7) ^a,b^	2.4 (2.3, 2.5) ^b^	2.4 (2.2, 2.7) ^a,b^
Vitamin E (mg/day)	9.4 (9.2, 9.6) ^a^	9.2 (9.0, 9.3) ^a,b^	8.9 (8.5, 9.4) ^a,b^	8.6 (8.2, 9.0) ^b^	8.8 (8.1, 9.5) ^a,b^
Iron (mg/day)	9.1 (9.0, 9.3)^a^	9.0 (8.9, 9.1) ^a^	9.1 (8.8, 9.4) ^a^	8.6 (8.3, 8.8) ^b^	8.7 (8.1, 9.2) ^a,b^
Zinc (mg/day)	7.0 (6.9, 7.1) ^a^	6.7 (6.7, 6.8) ^b^	6.6 (6.4, 6.9) ^a,b,c^	6.2 (6.0, 6.4) ^c^	6.4 (6.0, 6.9) ^a,b,c^
Selenium (µg/day)	59.1 (58.1, 60.2) ^a^	57.6 (56.9, 58.3) ^a,b^	56.4 (54.0, 58.8) ^a,b^	54.9 (53.1, 56.8) ^b^	55.5 (51.8, 59.3) ^a,b^
Age 13 years					
*n*	1152	2675	239	344	84
Macronutrients					
Energy (kJ/day)	8224 (8101, 8346)	8229 (8147, 8310)	8370 (8067, 8673)	8270 (8034, 8505)	8229 (7729, 8728)
Male	9004 (8824, 9184)	8985 (8866, 9103)	9199 (8770, 9628)	9062 (8739, 9386)	9106 (8330, 9881)
Protein (g/day)	70.3 (69.2, 71.5) ^a^	69.1 (68.4, 69.9) ^a,b^	68.7 (65.9, 71.5) ^a,b^	66.8 (64.7, 69.0) ^b^	64.9 (60.9, 68.8) ^a,b^
Fat (g/day)	78.6 (77.1, 80.0	77.3 (76.3, 78.2)	78.9 (75.6, 82.2)	77.3 (74.4, 80.14)	75.8 (70.1, 81.5)
Free sugar (% energy)	16.1 (15.8, 16.5) ^a^	16.9 (16.7, 17.1) ^b^	17.2 (16.3, 18.1) ^a,b^	17.9 (17.1, 18.7) ^b^	17.9 (16.5, 19.4) ^a,b^
Dietary fibre (g NSP/day)	12.9 (12.7, 13.2)	13.0 (12.8, 13.2)	12.8 (12.2, 13.3)	12.2 (11.7, 12.7)	12.1 (11.0, 13.2)
Micronutrients					
Vitamin A					
Carotene (µg/day)	2476 (2353, 2598)	2350 (2270, 2430)	2372 (2112, 2632)	2216 (1999, 2433)	2010 (1603, 2417)
Retinol equivalents (µg RE/day)	771 (743, 799)	741 (720, 762)	741 (677, 806)	703 (654, 753)	660 (583, 737)
Niacin (mg NEq/day)	32.6 (32.1, 33.2)	32.4 (32.0, 32.8)	32.0 (30.7, 33.3)	31.6 (30.5, 32.7)	31.7 (29.5, 33.9)
Vitamin B_6_ (mg/day)	2.1 (2.0, 21)	2.0 (1.99, 2.0)	1.9 (1.8, 2.0)	2.0 (1.9, 2.0)	2.0 (1.8, 2.1)
Vitamin B_12_ (µg/day)	4.5 (4.3, 4.6)	4.3 (4.2, 4.4)	4.1 (3.9, 4.4)	4.2 (4.0, 4.5)	4.0 (3.5, 4.4)
Folate (µg/day)	232 (227, 237)	234 (230, 238)	225 (213, 237)	232 (222, 242)	227 (209, 244)
Vitamin D (µg/day)	2.7 (2.7, 2.8)	2.6 (2.6, 2.7)	2.7 (2.5, 2.9)	2.5 (2.3, 2.7)	2.6 (2.3, 2.9)
Vitamin E (mg/day)	9.3 (9.1, 9.6)	9.1 (9.0, 9.3)	9.2 (8.6, 9.8)	8.8 (8.4, 9.3)	9.4 (8.5, 10.4)
Iron (mg/day)	10.0 (9.8, 10.2)	10.1 (9.8, 10.2	10.1 (9.7, 10.6)	9.9 (9.5, 10.2)	9.6 (8.9, 10.2)
Zinc (mg/day)	7.7 (7.5, 7.8)	7.6 (7.4, 7.7)	7.4 (7.1, 7.6)	7.3 (7.0, 7.5)	6.9 (6.4, 7.6)
Selenium (µg/day)	63.5 (61.9, 65.1)	62.2 (61.3, 63.2	65.8 (61.6, 70.1)	59.9 (57.2, 63.6)	65.2 (58.9, 71.6)

NSP, non-starch polysaccharide. Data at age 7 years are shown in Taylor et al. (2016) [23]. Values are mean (95% confidence interval). ^a,b,c^ Values in the same row with unlike superscript letters are significantly different for each age point (*p* < 0.05). Additional nutrients for which there were no significant differences at both 10 and 13 years (data not shown): energy for girls, carbohydrate, retinol, thiamin, riboflavin, vitamin C, calcium, iodine.

**Table 4:** Food and food group intakes (g/day) from 3-day food records at age 10 and 13 years in children enrolled in ALSPAC who were classified into longitudinal picky eating (PE) categories between 2 and 5.5 years of age.

Food/Food Group	Never PE	Low PE	High PE		
			Early PE		Late PE
			Non-Persistent PE	Persistent PE	
Age 10 years					
*n*	1380	3159	275	402	98
Total meat	109 (106, 113) ^a^	101 (99, 103) ^b^	98 (90, 105) ^a,b^	83 (77, 89) ^c^	93 (82, 104) ^a,b,c^
Meat, carcass ^1^	62 (59, 65) ^a^	56 (54, 58) ^b^	51 (45, 57) ^b,c^	39 (35, 44) ^c^	43 (33, 53) ^b,c^
Processed meat ^2^	47 (45, 49) ^a^	45 (43, 46) ^a^	47 (42, 53) ^a^	44 (40, 48) ^a^	50 (42, 58) ^a^
Eggs and egg dishes	10 (9, 11)	9 (9, 10)	8 (6, 10.8)	8 (6, 9)	5 (3, 8)
Potatoes, plain or mashed	36 (34, 39) ^a^	33 (32, 35) ^a^	34 (28, 39) ^a^	23 (19, 27) ^b^	27 (18, 36) ^a,b^
Total vegetables	80 (77, 83) ^a^	68 (66, 70) ^b^	60 (54, 66) ^b,c^	41 (37, 46) ^d^	46 (35, 57) ^c,d^
Total fruit	75 (71, 79) ^a^	69 (67, 72) ^a^	64 (55, 73) ^a,b^	63 (55, 71) ^a,b^	53 (40, 66) ^b^
Total milk ^3^	216 (207, 225) ^a^	210 (204, 216) ^a^	224 (202, 246) ^a^	236 (214, 258) ^a^	231 (202, 246) ^a^
Total sweet foods	128 (124, 132	127 (125, 130	124 (116, 133)	124 (118, 130)	137 (121, 153)
Buns, cakes and pastries	28 (26, 29)	28 (27, 29)	28 (24, 31)	26 (23, 29)	27 (21, 32)
Fruit juice	113 (105, 121)	123 (118, 129)	124 (105, 143)	120 (103, 136)	118 (88, 148)
Sweet biscuits and cookies	19 (18, 20) ^a^	19 (18, 20) ^a^	20 (17, 22) ^a,b^	23 (20, 25) ^b^	21 (18, 25) ^a,b^
Chocolate confectionery	17 (16, 18) ^a^	19 (18, 19) ^a,b^	20 (17, 22) ^a,b^	21 (19, 24) ^b^	18 (15, 22) ^a,b^
Carbonated drinks with sugar	101 (92, 106)	104 (98, 109)	117 (96, 139)	103 (88, 117)	77 (54, 101)
Savoury sauces ^5^	16 (15, 17) ^a^	14 (13, 15) ^b^	13 (11, 16) ^a,b,c^	10 (8, 12) ^c^	11 (8, 14) ^a,b,c^
Age 13 years					
*n*	1152	2675	239	344	84
Total meat	127 (122, 131) ^a^	122 (118, 125) ^a^	121 (111, 130) ^a,b^	106 (96, 116) ^b^	93 (79, 107) ^b^
Meat, carcass ^1^	77 (73, 81) ^a^	74 (71, 77) ^a^	73 (64, 82) ^a,b^	65 (57, 74) ^a,b^	51 (40, 61) ^b^
Processed meat ^2^	50 (47, 53) ^a^	48 (46, 50) ^a,b^	48 (41, 55) ^a,b^	41 (36, 46) ^b^	42 (33, 51) ^a,b^
Eggs and egg dishes	10 (9, 11)	10 (9, 11)	8 (5, 11)	7 (5, 9)	9 (5, 14)
Potatoes, plain or mashed	42 (39, 45) ^a^	39 (36, 41) ^a,b^	39 (31, 46) ^a,b^	31 (25, 36) ^b^	29 (20, 38) ^a,b^
Total vegetables	96 (91, 101) ^a^	86 (83, 88) ^b^	76 (68, 84) ^b,c^	61 (54, 68) ^c^	55 (43, 67) ^c^
Total fruit	83 (77, 88) ^a^	80 (76, 83) ^a^	74 (63, 86) ^a,b^	64 (54, 73) ^b^	58 (43, 73) ^b^
Total milk ^3^	224 (211, 237) ^a^	215 (207, 223) ^a^	204 (179, 228) ^a^	239 (214, 264) ^a^	216 (178, 253) ^a^
Total sweet foods	106 (101, 110)	107 (104, 110)	117 (105, 128)	113 (104, 121)	115 (97, 132)
Buns, cakes and pastries	27 (24, 29)	27 (26, 29)	32 (26, 37)	25 (21, 29)	25 (17, 33)
Fruit juice	150 (138, 161) ^a^	172 (164, 180) ^b^	170 (141, 198) ^a,b^	177 (154, 200) ^a,b^	160 (116, 203) ^a,b^
Sweet biscuits and cookies	19 (17, 20)	18 (17, 19)	18 (15, 21)	23 (20, 26)	25 (18, 32)
Chocolate confectionery	15 (14, 16) ^a^	17 (16, 18) ^a^	18 (15, 22) ^a,b^	21 (18, 25) ^b^	17 (12, 22) ^a,b^
Carbonated drinks with sugar	124 (113, 136	130 (121, 138)	119 (93, 145)	144 (117, 171)	121 (79, 163)
Savoury sauces ^4^	18 (17, 19) ^a^	16 (16, 17) ^a^	17 (14, 10) ^a^	12 (10, 14) ^b^	12 (8, 16) ^a,b^

Values are mean (95% confidence interval). Data at age 7 years are shown in Taylor et al. (2016) [23]. ^a,b,c,d^ Values in the same row with unlike superscript letters are significantly different for each age point (*p* < 0.05). ^1^ Foods included carcass meat from lamb, pork, beef, poultry, liver and kidney. ^2^ Foods included sausages, ham, bacon, burgers, meat pies, breaded poultry, salami, etc. ^3^ Milks included whole, semi-skimmed, skimmed cows’ milk, other animal milks, soya milk, formula milk and cream. ^4^ Savoury sauces included gravy, salad dressing, barbecue sauce, etc.

## Supplementary Materials

The following are available online at https://www.mdpi.com/2072-6643/11/4/807/s1. Text S1: Food groups assessed at 3 years of age by 3-day food record that are not different between the picky eating groups, Table S1: Percentage of misreporting of energy intake in participants enrolled in ALSPAC who were classified into cross-sectional picky eating categories at age 3 years and/or into longitudinal picky eating categories at 2–5.5 years of age based on 3-day food records, Table S2: Nutrients intake from 3-day food records at age 10 and 13 years in children with plausible energy intakes enrolled in ALSPAC who were classified in to picky eating categories at age 3 years (cross-sectional), Table S3: Food and food group intakes (g/day) at age 10 and 13 years in children with plausible energy intakes enrolled in ALSPAC who were classified in to picky eating categories at age 3 years (cross-sectional) from 3-day food records, Table S4: Nutrient intakes from 3-day food records at age 10 and 13 years in children enrolled in ALSPAC with plausible energy intakes who were classified into longitudinal picky eating (PE) categories between 2 and 5.5 years of age, Table S5: Food and food group intakes (g/day) from 3-day food records at age 10 and 13 years in children enrolled in ALSPAC with plausible energy intakes who were classified into longitudinal picky eating categories between 2 and 5.5 years of age.
