# Supplementary material for: Diet at Age 10 and 13 Years in Children Identified as Picky Eaters at Age 3 Years and in Children Who Are Persistent Picky Eaters in A Longitudinal Birth Cohort Study
Source: Nutrients. 2019 Apr 10;11(4):807. doi: 10.3390/nu11040807 (PMC6521015; doi:10.3390/nu11040807)
Supplement: Supplementary file 1 [file nutrients-11-00807-s001.pdf]

Supplementary text S1

Food groups assessed at 3 years of age by 3-day food record that are not different between the picky eating groups:

High fibre breakfast cereals; other breakfast cereals; yoghurt; puddings and ice cream; buns, cakes, pastries; sugar confectionery; sugar, preserves, sweet spreads; crisps, savoury snacks; bread, all types; fat spreads, all types; potatoes, all types; pasta, rice, pizza; baked beans; eggs, egg dishes; cheese; nuts; milk- and tomato-based sauces; soup; sugar-sweetened dilutable drinks; diet dilutable drinks; tea; coffee

Supplementary Table S1 Percentage of misreporting of energy intake in participants enrolled in ALSPAC who were classified into cross-sectional picky eating categories at age 3 years and/or into longitudinal picky eating categories at 2–5.5 years of age based on 3-day food records

	n	Under-reporters (%)	Plausible reporters (%)	Over-reporters (%)
**Cross-sectional classification**				
Age 10 years				
Non-picky	2341	37.7	59.6	2.7
Somewhat picky	2169	34.7	62.1	3.2
Very picky	804	31.7	64.8	3.5
Total	5314	35.5	61.4	3.0
Age 13 years				
Non-picky	1981	63.4	35.5	1.1
Somewhat picky	1820	59.4	39.7	0.8
Very picky	693	58.1	39.9	1.9
Total	4494	61.1	37.9	1.1
**Longitudinal classification**				
Age 10 years				
Never	1380	37.3	59.5	3.3
Low	3159	35.6	61.5	2.9
High				
Early: Non-persistent	275	30.7	65.3	4.0
Early: Persistent	402	32.9	63.8	3.2
Late	98	31.6	67.3	1.0
Total	5314	35.5	61.4	3.0
Age 13 years				
Never	1152	64.6	34.7	0.7
Low	2675	60.1	38.8	1.1
High				
Early: Non-persistent	239	56.2	41.7	2.1
Early: Persistent	344	60.6	37.4	2.1
Late	84	53.6	45.2	1.2
Total	4494	61.0	37.9	1.1

For details of misreporting classification see Methods in main text.

Supplementary Table S2 Nutrients intake from 3-day food records at age 10 and 13 years in children with plausible energy intakes enrolled in ALSPAC who were classified in to picky eating categories at age 3 years (cross-sectional)

Nutrient	Age 10 years			Age 13 years		
	Non-picky eater	Somewhat picky eater	Very picky eater	Non-picky eater	Somewhat picky eater	Very picky eater
n	1386	1340	520	696	717	272
Macronutrients						
Energy (MJ)	8.50 (8.44, 8.57)	8.34 (8.28, 8.40)**	8.28 (8.18, 8.38)**	9.98 (9.86, 10.10)	9.81 (9.70, 9.93)	9.95 (9.76, 10.13)
Protein (g/day)	67.0 (66.3, 67.8)	64.9 (64.1, 65.6)***	63.2 (62.1, 64.4)***	81.1 (79.7, 82.5)	79.0 (77.6, 80.3)	78.4 (75.9, 80.8)
Free sugar (% energy)	17.8 (17.6, 18.1)	18.5 (18.2, 18.8)**	18.5 (18.0, 19.0)	17.6 (17.1, 18.)	18.3 (17.9, 18.7)	18.7 (17.9, 19.6)*
Dietary fibre (g/day)	12.4 (12.2, 12.6)	12.1 (11.9, 12.3)	11.5 (11.2, 11.8)***	14.9 (14.5, 15.2)	14.7 (14.3, 15.0)	14.1 (13.5, 14.7)
Micronutrients						
Vitamin A						
Carotene (µg/day)	2209 (2123, 2294)	2189 (2096, 2281)	1940 (1790, 2089)**	2649 (2490, 2808)	2542 (2384, 2701)	2543 (2324, 2762)
Vitamin B_{12} (µg/day)	3.77 (3.66, 3.87)	3.66 (3.56, 3.75)	3.60 (3.41, 3.79)	5.14 (4.95, 5.32)	4.86 (4.69, 5.03)	4.84 (4.56, 5.12)
Vitamin D (µg/day)	2.85 (2.78, 2.92)	2.82 (2.74, 2.90)	2.63 (2.53, 2.74)**	3.25 (3.12, 3.39)	3.08 (2.97, 3.20)	3.11 (2.93, 3.29)
Vitamin E (mg/day)	9.96 (9.77, 10.15)	9.75 (9.56, 9.94)	9.37 (9.04, 9.70)**	11.2 (10.8, 11.5)	10.6 (10.3, 10.9)*	10.7 (10.2, 11.3)
Zinc (mg/day)	7.4 (7.2, 7.5)	7.1 (7.0, 7.2)**	6.8 (6.7, 7.0)***	8.9 (8.7, 9.1)	8.7 (8.5, 8.9)	8.4 (8.1, 8.7)*
Iron (mg/day)	9.6 (9.5, 9.8)	9.6 (9.5, 9.7)	9.4 (9.2, 9.6)	11.8 (11.5, 12.0)	11.7 (11.4, 11.9)	11.5 (11.1, 11.9)
Selenium (µg/day)	62.0 (61.0, 63.0)	60.9 (59.8, 61.9)	59.2 (57.5, 60.9)*	72.8 (70.3, 75.3)	71.2 (69.4, 73.0)	70.9 (67.2, 74.6)

Values are mean (95% confidence interval).

Value at same age significantly different from that of children who were non-picky eaters: *$p \le 0.05$, **$p \le 0.01$, ***$p \le 0.001$.

Additional nutrients for which there were no significant differences at both 10 and 13 years (data not shown): carbohydrate, fat, retinol, retinol equivalents, thiamin, riboflavin, niacin, vitamin B_6, folate, vitamin C, calcium, iodine.

See Supplementary Table 1 for details of classification of plausible energy intakes.

Supplementary Table S3 Food and food group intakes (g/day) at age 10 and 13 years in children with plausible energy intakes enrolled in ALSPAC who were classified in to picky eating categories at age 3 years (cross-sectional) from 3-day food records

	**Age 10 years**			**Age 13 years**		
	**Non-picky eater**	**Somewhat picky eater**	**Picky eater**	**Non-picky eater**	**Somewhat picky eater**	**Picky eater**
n	1386	1340	520	696	717	272
Total meat	112 (109, 115)	100 (97, 103)***	95 (90, 100)***	139 (132, 145)	131 (124, 137)	130 (119, 141)
Meat, carcass [1]	63 (60, 66)	56 (53, 59)**	47 (43, 51)***	82 (76, 87)	79 (74, 85)	80 (70, 90)
Processed meat [2]	49 (47, 51)	44 (42, 46)*	48 (44, 52)	57 (53, 55)	51 (47, 57)	50 (43, 56)
Total fish	14 (13, 15)	15 (14, 16)	12 (10, 14)	17 (15, 20)	16 (14, 19)	14 (10, 18)
Total vegetables	76 (73, 80)	69 (66, 72)**	53 (49, 57)***	98 (93, 104)	90 (84, 95)	76 (68, 85)***
Total fruit	76 (72, 80)	74 (70, 78)	70 (63, 77)	94 (86, 103)	95 (88, 103)	81 (70, 93)
Total milk [3]	241 (231, 251)	231 (221, 241)	250 (232, 268)	275 (255, 294)	249 (233, 265)	263 (234, 293)
Total sweet foods	143 (140, 147)	144 (141, 148)	135 (130, 141)	149 (142, 155)	146 (140, 151)	153 (143, 163)
Chocolate confectionery	20 (19, 21)	22 (21, 23)	23 (21, 25)*	21 (19, 23)	23 (21, 25)	23 (20, 26)
Buns, cakes, and pastries	32 (30, 34)	32 (31, 34)	29 (27, 32)	41 (38, 45)	40 (37, 43)	41 (35, 47)
Sweet biscuits and cookies	22 (21, 23)	21 (20, 22)	22(20, 23)*	26(24, 29)	25 (23, 27)	23 (20, 26)
Soft drinks with sugar	117 (108, 126)	109 (100, 118)	111 (97, 124)	164 (146, 182)	157 (138, 176)	171 (138, 204)
Fruit juice	124 (116, 132)	139 (130, 148)*	129 (125, 137)	183 (166, 199)	209 (192, 226)	219 (192, 245)
Savoury sauces [4]	16 (15, 17)	14 (13, 15)**	12 (11, 14)***	20 (18, 22)	17 (15, 19)*	17 (14, 19)

Values are mean (95% confidence interval).

Value at same age significantly different from that of children who were not picky eaters: *p≤0.05, **p≤0.01, ***p≤0.001.

[1]Foods included carcass meat from lamb, pork, beef, poultry, liver and kidney.

[2]Foods included sausages, ham, bacon, burgers, meat pies, breaded poultry, salami, etc.

[3]Milks included whole, semi-skimmed, skimmed cows' milk, other animal milks, soya milk and cream.

[4]Savoury sauces included gravy, salad dressing, barbecue sauce, etc.

See Supplementary Table 1 for details of classification of plausible energy intakes.

Supplementary Table S4 Nutrient intakes from 3-day food records at age 10 and 13 years in children enrolled in ALSPAC with plausible energy intakes who were classified into longitudinal picky eating (PE) categories between 2 and 5.5 years of age

Nutrient	Never PE	Low PE	High PE		
			Early PE		Late PE
			Non-persistent PE	Persistent PE	
**Age 10 years**					
n	814	1931	179	256	66
Macronutrients					
Energy (MJ/day)	8.58 (8.50, 8.67)[a]	8.35 (8.30, 8.41)[b]	8.37 (8.19, 8.54)[ab]	8.24 (8.10, 8.39)[b]	8.14 (7.84, 8.43)[b]
Protein (g/day)	67.9 (66.9, 68.8)[a]	65.2 (64.6, 65.9)[b]	64.0 (62.0, 66.0)[bc]	62.0 (60.3, 63.8)[c]	62.6 (59.3, 66.0)[bc]
Fat (g/day)	84.0 (82.8, 85.3)[a]	80.9 (80.2, 81.6)[b]	81.4 (79.0, 83.9)[ab]	79.6 (77.4, 81.8)[b]	77.4 (77.4, 81.9)[b]
Free sugar (% energy)	17.7 (17.3, 18.1)[a]	18.3 (18.1, 18.6)[b]	18.3 (17.4, 19.1)[ab]	18.7 (18.0, 19.4)[ab]	19.5 (18.0, 20.9)[ab]
Dietary fibre (g NSP/day)	12.6 (12.4, 12.8)[a]	12.2 (12.0, 12.3)[b]	11.6 (11.1, 12.1)[bc]	11.1 (10.7, 11.5)[c]	11.4 (10.6, 12.1)[bc]
Micronutrients					
Vitamin A					
Carotene (µg/day)	2301 (2186, 2417)[a]	2170 (2094, 2245)[a]	2121 (1890, 2312)[ab]	1694 (1472, 1917)[b]	1913 (1542, 2284)[ab]
Retinol equivalents (µg RE/day)	782 (752, 812)[a]	745 (722, 768)[a]	818 (704, 932)[a]	637 (595, 679)[b]	764 (534, 994)[ab]
Niacin (mg NEq/day)	31.7 (31.2, 32.2)[a]	30.9 (30.6, 31.2)[ab]	30.6 (29.6, 31.7)[ab]	29.9 (29.0, 30.8)[b]	29.7 (27.9, 31.5)[ab]
Vitamin B_6 (mg/day)	2.0 (2.0, 2.1)	2.0 (1.9, 2.0)	2.0 (1.9, 2.1)	1.9 (1.8, 1.9)	1.9 (1.7, 2.0)
Vitamin B_{12} (µg/day)	3.7 (3.6, 3.9)	3.7 (3.6, 3.8)	3.7 (3.3, 4.2)	3.4 (3.2, 3.6)	3.8 (3.1, 4.5)
Folate (µg/day)	226.0 (221.4, 230.6)	221.8 (218.7, 224.9)	225.6 (215.3, 235.8)	215.2 (206.2, 224.2)	201.7 (185.8, 217.7)
Vitamin D (µg/day)	2.9 (2.8, 3.0)	2.8 (2.8, 2.9)	2.6 (2.6, 2.8)	2.6 (2.5, 2.8)	2.5 (2.2, 2.8)
Vitamin E (mg/day)	10.0 (9.8, 10.3)	9.8 (9.6, 9.9)	9.4 (8.9, 9.9)	9.4 (8.9, 9.9)	9.0 (8.2, 9.9)
Iron (mg/day)	9.7 (9.6, 9.8)[a]	9.6 (9.5, 9.7)[ab]	9.4 (9.1, 9.8)[ab]	9.2 (9.0, 9.5)[b]	9.3 (8.7, 10.0)[ab]
Zinc (mg/day)	7.4 (7.3, 7.6)[a]	7.2 (7.1, 7.2)[b]	6.9 (6.6, 7.1)[bc]	6.7 (6.4, 7.0)[c]	6.9 (6.3, 7.4)[abc]
Selenium (µg/day)	62.6 (61.3, 63.8)[a]	61.1 (60.3, 62.0)[ab]	58.5 (55.6, 61.4)[ab]	58.4 (56.3, 60.7)[b]	57.3 (52.6, 61.9)[ab]
**Age 13 years**					
n	394	1028	98	127	38
Macronutrients					
Energy (kJ/day)	10.06 (9.90, 10.23)	9.84 (9.74, 9.93)	10.05 (9.70, 10.41)	9.86 (9.62, 10.09)	9.93 (9.36, 10.50)
Protein (g/day)	81.9 (80.0, 83.9)	79.5 (78.4, 80.6)	79.4 (74.9, 83.8)	77.0 (73.7, 80.4)	74.1 (68.0, 80.3)
Fat (g/day)	98.6 (96.5, 100.6)[a]	93.7 (92.5, 95.0)[b]	96.0 (92.1, 99.9)[ab]	94.7 (91.3, 98.0)[ab]	92.2 (84.3, 100.0)[ab]
Free sugar (% energy)	17.1 (16.5, 17.7)[a]	18.2 (17.8, 18.6)[b]	18.6 (17.1, 20.1)[ab]	18.7 (17.5, 19.8)[ab]	20.6 (18.6, 22.6)[b]
Dietary fibre (g NSP/day)	15.0 (14.5, 15.4)[a]	14.7 (14.4, 15.0)[a]	14.6 (13.7, 15.5)[ab]	13.4 (12.6, 14.2)[b]	15.1 (13.4, 16.8)[ab]
Micronutrients					
Vitamin A					
Carotene (µg/day)	2736 (2516, 2957)	2568 (2439, 2698)	2649 (2291, 3008)	2297 (1980, 2615)	2333 (1606, 3061)
Retinol equivalents (µg RE/day)	923 (870, 975)	845 (812, 877)	869 (759, 980)	826 (732, 920)	773 (642, 905)
Niacin (mg NEq/day)	37.2 (36.2, 38.1)	37.0 (36.4, 37.6)	36.2 (34.3, 38.1)	35.8 (34.0, 37.6)	37.2 (33.9, 40.6)
Vitamin B_6 (mg/day)	2.3 (2.3, 2.4)	2.3 (2.3, 2.3)	2.2 (2.1, 2.4)	2.2 (2.1, 2.3)	2.3 (2.1, 2.6)
Vitamin B_{12} (µg/day)	5.2 (4.9, 5.4)	4.9 (4.8, 5.1)	4.4 (4.0, 4.8)	5.1 (4.6, 5.6)	4.3 (3.5, 5.0)
Folate (µg/day)	266.5 (257.6, 275.4)	267.6 (261.9, 273.2)	260.0 (242.6, 277.4)	268.5 (252.4, 284.6)	252.2 (225.9, 278.5)
Vitamin D (µg/day)	3.3 (3.1, 3.4)	3.1 (3.0, 3.2)	3.1 (2.8, 3.4)	3.1 (2.8, 3.3)	2.9 (2.4, 3.7)
Vitamin E (mg/day)	11.5 (11.1, 11.9)[a]	10.6 (10.4, 10.9)[b]	11.1 (10.2, 12.0)[ab]	10.6 (9.9, 11.3)[ab]	10.8 (9.2, 12.3)[ab]
Iron (mg/day)	11.8 (11.5, 12.1)	11.7 (11.5, 11.9)	11.7 (11.1, 12.3)	11.2 (10.7, 11.8)	11.5 (10.6, 12.5)
Zinc (mg/day)	9.0 (8.7, 9.2)	8.7 (8.6, 8.9)	8.4 (8.0, 8.8)	8.3 (7.9, 8.7)	8.0 (7.3, 8.7)
Selenium (µg/day)	73.1 (69.5, 76.7)	71.2 (69.6, 72.8)	76.3 (68.3, 84.3)	68.1 (63.6, 72.5)	75.8 (65.0, 86.6)

NSP, non-starch polysaccharide.

Data at age 7 years are shown in Taylor et al. (2016).

Values are mean (95% confidence interval).

[a,b,c] Values in the same row with unlike superscript letters are significantly different for each age point ($p<0.05$).

Additional nutrients for which there were no significant differences at both 10 and 13 years (data not shown): energy for girls, carbohydrate, retinol, thiamin, riboflavin, vitamin C, calcium, iodine.

See Supplementary Table 1 for details of classification of plausible energy intakes.

Supplementary Table S5 Food and food group intakes (g/day) from 3-day food records at age 10 and 13 years in children enrolled in ALSPAC with plausible energy intakes who were classified into longitudinal picky eating categories between 2 and 5.5 years of age

Food/food group	Never PE	Low PE	High PE		
			Early PE		Late PE
			Non-persistent PE	Persistent PE	
**Age 10 years**					
n	814	1931	179	256	66
Total meat	114 (110, 119)[a]	103 (101, 106)[b]	99 (90, 108)[bc]	89 (81, 97)[c]	94 (82, 107)[abc]
Meat, carcass [1]	65 (61, 68)[a]	58 (55, 60)[b]	52 (44, 59)[b]	43 (36, 49)[b]	44 (32, 56)[b]
Processed meat [2]	50 (47, 53)	46 (44, 48)	47 (41, 53)	46 (40, 52)	50 (42, 59)
Eggs and egg dishes	10 (9, 11)	10 (9, 11)	8 (5, 10)	7 (5, 9)	4 (2, 7)
Potatoes, plain or mashed	40 (37, 43)	35 (33, 37)	37 (30, 44)	22 (18, 27)	26 (15, 37)
Total vegetables	80 (76, 84)[a]	71 (68, 73)[b]	62 (54, 69)[b]	42 (36, 47)[c]	48 (37, 59)[bc]
Total fruit	78 (72, 83)	74 (71, 77)	75 (63, 88)	69 (59, 79)	58 (42, 74)
Total milk [3]	238 (226, 252)	235 (226, 243)	232 (206, 258)	265 (235, 295)	245 (192, 299)
Total sweet foods	146 (141, 151)	142 (139, 145)	138 (127, 149)	136 (128, 143)	151 (131, 171)
Buns, cakes and pastries	32 (30, 35)	32 (31, 34)	31 (26, 35)	30 (25, 34)	31 (24, 38)
Fruit juice	119 (108, 130)	136 (129, 144)	134 (110, 158)	126 (105, 147)	130 (90, 170)
Sweet biscuits and cookies	22 (20, 23)	21 (20, 22)	22 (19, 26)	23 (20, 26)	22 (17, 27)
Chocolate confectionery	20 (19, 22)	21 (20, 22)	23 (19, 26)	24 (21, 26)	20 (15, 25)
Soft drinks with sugar	113 (101, 124)	114 (106, 121)	117 (92,142)	106 (86, 126)	88 (58, 118)
Savoury sauces [5]	17 (16, 18)[a]	15 (14, 15)[b]	13 (10, 16)[abc]	10 (8, 11)[c]	12 (8, 16)[abc]
**Age 13 years**					
n	394	1028	98	127	38
Total meat	138 (130, 147)	135 (130, 140)	135 (119, 150)	117 (99, 136)	108 (80, 133)
Meat, carcass [1]	84 (77, 91)	80 (76, 85)	84 (69, 100)	75 (59, 90)	59 (39, 79)
Processed meat [2]	54 (49, 59)	55 (51, 58)	50 (38, 63)	43 (34, 51)	48 (33, 63)
Eggs and egg dishes	12 (10, 15)	11 (10, 13)	9 (4, 13)	8 (4, 11)	7 (2, 13)
Potatoes, plain or mashed	49 (43, 56)[a]	40 (37, 43)[ab]	37 (26, 48)[ab]	30 (22, 38)[b]	32 (17, 48)[ab]
Total vegetables	106 (98, 114)[a]	91 (86, 96)[b]	86 (72, 100)[ab c]	61 (51, 72)[c]	62 (42, 83)[bc]
Total fruit	97 (86, 108)[a]	95 (88, 101)[a]	105 (83, 127)[ac]	64 (49, 79)[b]	64 (41, 87)[abc]
Total milk [3]	280 (253, 308)[a]	257 (23, 271)[a]	184 (151, 216)[b]	313 (262, 365)[ac]	227 (167, 187)[abc]
Total sweet foods	152 (144, 161)	146 (141, 151)	157 (140, 173)	143 (129, 157)	160 (131, 189)
Buns, cakes and pastries	44 (39, 48)	40 (37, 42)	47 (36, 59)	34 (27, 41)	39 (25, 52)
Fruit juice	180 (158, 202)	204 (190, 218)	235 (183, 287)	203 (169, 238)	198 (122, 273)
Sweet biscuits and cookies	27 (24, 31)	24 (23, 26)	24 (19, 28)	26 (22, 33)	34 (20, 48)
Chocolate confectionery	20 (18, 23)	22 (21, 24)	22 (17, 27)	27 (22, 32)	23 (13, 32)
Soft drinks with sugar	153 (131, 175)	164 (148, 180)	148 (102, 194)	184 (127, 241)	180 (102, 257)
Savoury sauces [4]	20 (17, 22)[a]	18 (17, 20)[ab]	21 (15, 26)[ab]	12 (9, 15)[b]	17 (10, 24)[ab]

Values are mean (95% confidence interval).

[a,b,c]Values in the same row with unlike superscript letters are significantly different for each age point ($p<0.05$).

[1]Foods included carcass meat from lamb, pork, beef, poultry, liver and kidney.

[2]Foods included sausages, ham, bacon, burgers, meat pies, breaded poultry, salami, etc.

[3]Milks included whole, semi-skimmed, skimmed cows' milk, other animal milks, soya milk and cream.

[4]Savoury sauces included gravy, salad dressing, barbecue sauce, etc.

See Supplementary Table 1 for details of classification of plausible energy intakes.
